# Wastewater treatment by algae-based membrane bioreactors: a review of the arrangement of a membrane reactor, physico-chemical properties, advantages and challenges

**DOI:** 10.1039/d4ra04417g

**Published:** 2024-10-30

**Authors:** Jayaprabakar Jayaraman, J. Kumaraswamy, Yarrapragada K. S. S. Rao, M. Karthick, S. Baskar, M. Anish, Abhishek Sharma, Anil Singh Yadav, Tabish Alam, Muhammad Imam Ammarullah

**Affiliations:** a Department of Mechanical Engineering, Sathyabama Institute of Science & Technology Chennai 600119 Tamil Nadu India; b Department of Mechanical Engineering, R. L. Jalappa Institute of Technology, Affiliated to Visvesvaraya Technological University (V.T.U) Belagavi 590018 Karnataka India; c Department of Mechanical Engineering, Aditya University Surampalem 533437 Andhra Pradesh India; d Department of Mechanical Engineering, Vel Tech Rangarajan Dr Sagunthala R&D Institute of Science and Technology Chennai 600062 Tamil Nadu India; e School of Engineering, Vels Institute of Science, Technology & Advanced Studies Chennai 600117 Tamil Nadu India; f Department of Mechanical Engineering, Government Engineering College (Department of Higher and Technical Education, Govt. of Jharkhand) Medininagar 822118 Jharkhand India drasharma58@gmail.com; g Department of Mechanical Engineering, Bakhtiyarpur College of Engineering (Science, Technology and Technical Education Department, Govt. of Bihar) Bakhtiyarpur Patna 803212 Bihar India; h Architecture Planning and Energy Efficiency, CSIR-Central Building Research Institute Roorkee 247667 Uttarakhand India; i Department of Mechanical Engineering, Faculty of Engineering, Universitas Diponegoro Semarang 50275 Central Java Indonesia; j Undip Biomechanics Engineering & Research Centre (UBM-ERC), Universitas Diponegoro Semarang 50275 Central Java Indonesia imamammarullah@gmail.com

## Abstract

Reducing wastewater contaminants is an emerging area of particular concern for many industrialized and developing countries in improving the ecological quality of their water sources. In this case, the use of algae-based microbial reactors for wastewater treatment has attracted increasing attention in recent years. The advantages of both conventional microbial membrane bioreactors (MBRs) and algae-based treatment are combined in algae-based MBRs. According to the literature, previous studies did not fully discuss the techniques and performance of algae-based bioreactor systems in the treatment of wastewater. In particular, little attention has been paid to the types of waste, their consequences, and the ways in which they are treated. This makes it more difficult to develop and scale up efficient systems to treat waste discharge from industry, agriculture, and urban areas. Thus, the objective of this study is to critically evaluate algae as a valuable biological resource for wastewater treatment, with the goal of reducing emerging contaminants and increasing the chemical oxygen demand (COD) in wastewater. The most common wastewater treatment techniques employed for addressing these wastes are examined together with a brief discussion on contaminants in wastewater. Furthermore, algae-based wastewater treatment arrangements, particularly hybrid configurations, are carefully studied in relation to techniques for removing contaminants using algae. After analysing the key physicochemical characteristics that affect the ability of algal-bioremediation to remove developing contaminants, the benefits of algal-bioremediation systems are compared to those of other techniques. Lastly, an investigation is conducted into the technological difficulties associated with employing algal-bioremediation systems to eliminate emerging contaminants.

## Abstract 2

Reducing wastewater contaminants is an emerging area of particular concern for many industrialized and developing countries in improving the ecological quality of their water sources.

## Graphical abstract

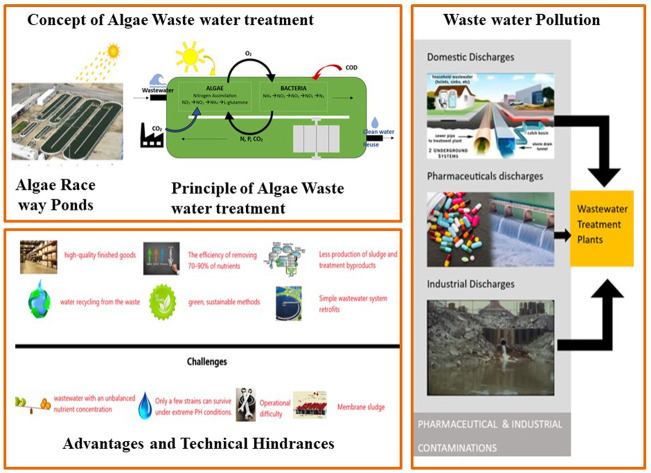

## 1 Introduction

**Fig. 1:**
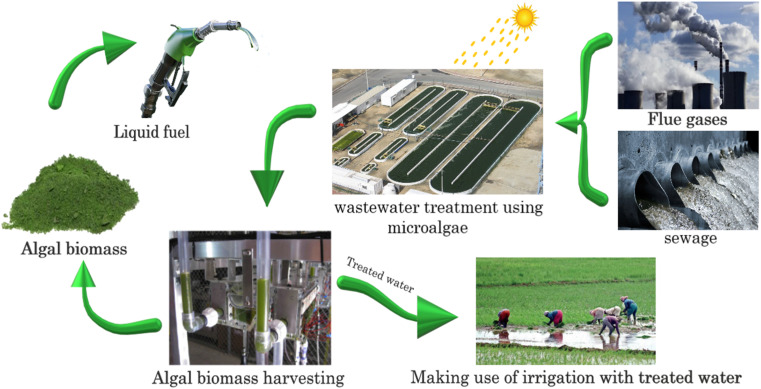
Typical algae-based wastewater treatment.

Developing and expanding effective systems for the treatment of waste discharge from industry, agriculture, and urban areas have become increasingly challenging.^1^ Wastewater treatment is a vital process that aims to remove pollutants and contaminants from wastewater before releasing it back into the environment.^2^ However, traditional wastewater treatment methods have several limitations, such as high energy consumption, chemical usage, and low efficiency.^3^ Alternatively, algae-based membrane bioreactors (MBRs) are a relatively new wastewater treatment technology that has shown promising results in recent years.^4^Fig. 1 depicts the typical algae-wastewater treatment process. Algae developed by feeding sewage water and flue gases can be harvested and converted into beneficial products such as liquid fuel, and finally, the treated water is suitable for irrigation.

Algae-based MBRs combine the benefits of algae-based treatment and MBRs. Algae are known for their ability to remove nutrients, such as nitrogen and phosphorus, from wastewater through photosynthesis.^5^ Alternatively, MBRs use a membrane to separate wastewater from biomass, resulting in higher treatment efficiency and less sludge production.^6^ Algae-based MBRs use a membrane to retain algae biomass, providing a stable environment for algal growth, while removing pollutants from wastewater.^7^ One of the key advantages of algae-based MBRs is their ability to remove pollutants from wastewater efficiently. Algae are effective in removing nutrients, organic matter, and some heavy metals from wastewater. They also have the ability to remove micropollutants, such as pharmaceuticals and personal care products, which are not efficiently removed *via* traditional wastewater treatment methods.^8^ Additionally, the membrane in MBRs provides a physical barrier to remove suspended solids, resulting in a higher-quality effluent. Another advantage of algae-based MBRs is their low energy consumption. Algae-based treatment does not require energy intensive aeration or chemical dosing, resulting in lower energy consumption than traditional wastewater treatment methods. Additionally, the use of membranes reduces the need for secondary clarification, which further reduces the energy consumption. However, despite the numerous advantages of algae-based MBRs, there are also several challenges associated with their implementation. One challenge is the high initial investment cost. The cost of constructing and operating an algae-based MBR system is currently higher than traditional wastewater treatment methods.^9^ Nevertheless, with the advancements in technology and the increasing demand for sustainable wastewater treatment, the cost of algae-based MBRs is expected to decrease in the future. Another problem associated with algae-based MBRs is the management of the algal biomass.^10^

**Fig. 2:**
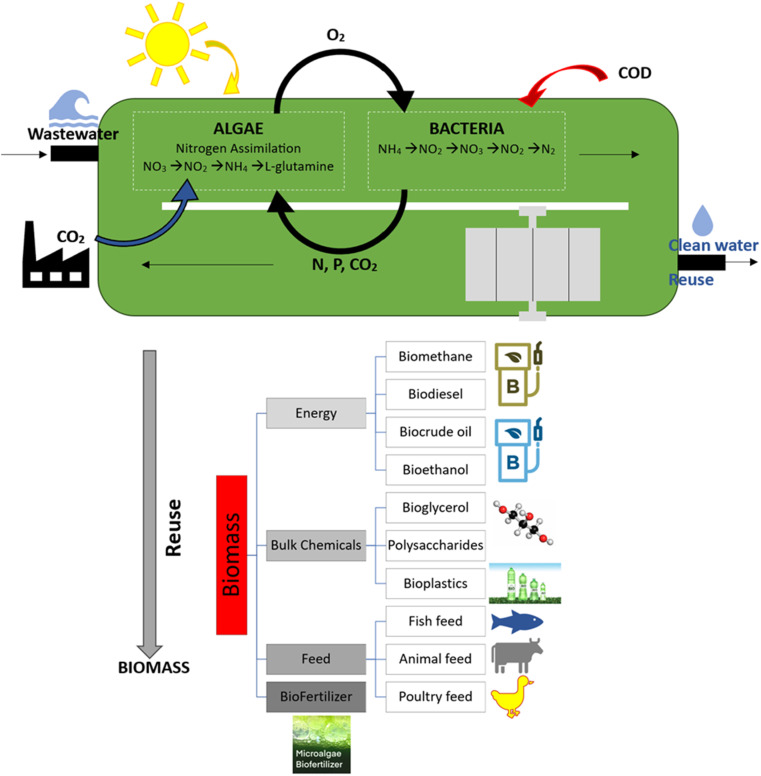
Algae biomass for wastewater treatment and reusing options.

The use of algal biomass for water treatment, options for reusing the leftover biomass and the key processes in the treatment of bacterial and algae-based wastewater are shown in Fig. 2.

The reactions involved in the nitrate reduction process during algal photosynthesis are given in eqn (1) and (2), as follows:

1 4H_2_O + NO_3_^−^ → 7OH^−^ + NH_4_^+^

2 CO_2_ + NH_4_^+^ + PO_4_^−3^ + H_2_O → O_2_ + microalgal biomass

The reactions during the denitrification and nitrification process involving bacteria are given in eqn (3)–(5), as follows:

3 5CH_3_COOH + 8NO_3_^−^ → 6H_2_O + 8HCO_3_^−^ + 4N_2_ + 2CO_2_

4 2O_2_ + NH_4_^+^ → NO_3_^−^ + H_2_O + 2H^+^

5 O_2_ + COD + nutrients → CO_2_ + bacterial biomass

The primary metabolic processes and the absorption of nitrogen by bacteria and algae are also displayed in Fig. 2. Because they have all the resources needed to carry out their metabolic processes, heterotrophic microalgae never stop growing. Microalgae can carry out conversion through a variety of cultivation modes, such as photoautotrophic metabolism, which occurs in an environment with light, water, and inorganic carbon; heterotrophic metabolism, occurring in the absence of light and involving the use of organic carbon (glucose, acetate); and mixotrophic cultivation, involving the simultaneous use of inorganic and organic carbon in the presence of light.^11^ Photoautotrophic microalgae are responsible for the assimilation and consumption of nitrogen, phosphorous, and CO_2_ in their dissolved forms. During the process of conversion, photosynthetic carbon fixation forms carbohydrates or lipids, while aerobic bacteria use the O_2_ produced as an electron acceptor. Photochemically fixed carbon dioxide (CO_2_) in the form of glucose is the only energy source available for the processes involved in the metabolism of the algae cells. Conversely, because autotrophic microalgae lose carbohydrates through respiration, they grow throughout the day and shrink at night. Aerobic heterotrophic bacteria in traditional biological treatment systems contribute to the breakdown of organic compounds by oxidizing or removing BOD, which releases CO_2_.^11^ In terms of energy involvement, algae-based reactors are the future in the development for efficient wastewater treatment technology with sustainability and efficiency. The algal biomass that remains after water treatment can be reused. Algal bio-fuel is not the only product that can be made from algae, where many other by-products from the processing process have a longer history of use. Fish and animal feed, bio-plastics, natural dyes and pigments, bio-fertilizers, antioxidants, and other high-value bio-active substances are a few of these products. However, algal biomass can accumulate on the membrane surface, reducing the efficiency of the system and potentially causing fouling. Additionally, the biomass produced can be challenging to dispose, especially if it contains high levels of nitrogen and phosphorus. Nevertheless, the advantages of algae-based MBRs, such as their low energy consumption and high treatment efficiency, make them a promising alternative to traditional wastewater treatment methods.^12^ In this case, the challenges associated with their implementation, such as high initial investment costs and biomass management, need to be addressed to make them more viable for widespread adoption.

The aim of this review is to summarize the knowledge in the field of wastewater treatment and provide methodological insight into the performance of the algae-based wastewater treatment method. The effectiveness of algal bioreactor systems for wastewater treatment is not entirely covered by prior research. Specifically, the types of waste, their effects, and the methods by which they are handled received very little consideration. Thus, this study aims to assess algae critically as a useful biological resource for treating wastewater to lower emergent pollutants and raise the COD of wastewater. Initially, various wastewater treatment technologies are discussed and their benefits and challenges explained. Subsequently, algae-based wastewater treatment approaches are briefly covered before moving on to a more in-depth examination of the physico-chemical factors influencing algal-bio-remediation. Finally, the benefits and drawbacks of using algae in wastewater treatment are discussed in relation to other current methods.

## 2 Emerging pollutants in wastewater

**Fig. 3:**
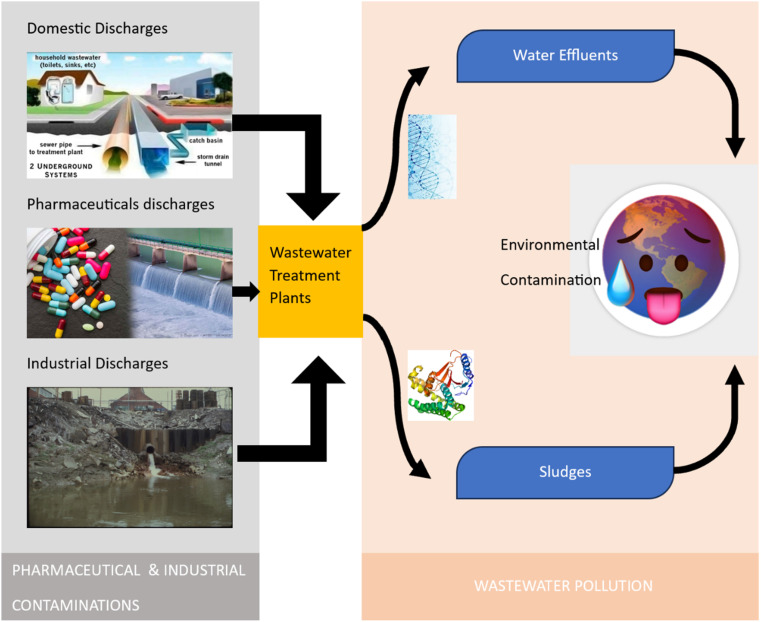
Sources of industrial and pharmaceutical contaminations.

Emerging pollutants are substances that are not commonly monitored or regulated, but are increasingly being recognized as potentially harmful to the environment and human health. In wastewater, emerging pollutants can come from a variety of sources including pharmaceuticals, personal care products, pesticides, industrial chemicals, and microplastics.^13^ The sources of industrial and pharmaceutical pollutants are grouped in Fig. 3.

Pharmaceuticals pollutants are one of the most common types of emerging pollutants found in wastewater. Many drugs are not completely metabolized by the body and are excreted. Consequently, they can end up in wastewater treatment plants, and ultimately the environment. Studies have shown that pharmaceuticals can have a variety of negative effects on aquatic organisms, including changes in their behaviour, growth, and reproduction.^14^ Personal care products such as lotions, shampoos, and soaps are also considered as emerging pollutants in wastewater.^15^ Many of these products contain chemicals that are designed to resist breakdown and can persist in the environment for long periods. Some of these chemicals, such as triclosan and phthalates, have been shown to have negative effects on aquatic organisms and may also be harmful to human health. Pesticides are another source of emerging pollutants in wastewater. These chemicals are used in agriculture to control pests and can find their way into wastewater through runoff. Pesticides have been shown to have negative effects on aquatic organisms, including changes in their behaviour, growth, and reproduction. Industrial chemicals are also pollutants, which include chemicals used in manufacturing, such as flame retardants and plasticizers, as well as chemicals used in everyday products such as electronics and furniture. Many of these chemicals are not biodegradable and can persist in the environment for long periods, potentially causing harm to aquatic organisms and humans. Microplastics are a type of emerging pollutant that has received increasing attention in recent years.^16^ Microplastics are small plastic particles that are less than 5 mm in size and can come from a variety of sources, including clothing, cosmetics, and packaging. These particles can persist in the environment for hundreds of years and have been shown to have negative effects on aquatic organisms, including changes in their behaviour, growth, and reproduction. Overall, the presence of emerging pollutants in wastewater is a growing concern due to their potential negative effects on both the environment and human health. However, although efforts are being devoted to better understanding and mitigating the impacts of these pollutants, more research is required to fully understand the extent of the problem and develop effective solutions.

### 2.1 Pollutants from treated wastewater

Wastewater treatment plants (WWTPs) with the conventional design cannot remove most chemicals from wastewater. Recipient bodies such as rivers, lakes, and sea waters may receive treated wastewater released by WWTPs. Consequently, a considerable amount of the substances present in wastewater effluents, together with their metabolites and transformation products have been discovered in surface waters and the marine environment, which is a significant concern for scientists. Nevertheless, a significant number of chemical substances are not eliminated by these methods. For instance, certain medications, such as paracetamol (99%) and ibuprofen (between 70% and 100%), are effectively eliminated from wastewater using traditional treatment techniques, whereas other medications, such sulfamethazine (13%) and carbamazepine (between 7% and 23%), are removed in much lower quantities.^17,18^ Furthermore, transformation products can arise from a variety of processes that occur in natural waters, including photo-degradation, hydrolysis, and biodegradation. These processes can also occur during wastewater treatment and water disinfection.^19,20^ Wastewater chemicals have the ability to break down and/or react with other compounds in the environment to release products that are more harmful than the original compounds. In this case, determining the hazardous effects of medications, their degradation products, and mixtures in the environment is a difficult task for scientists and a topic that needs immediate attention. Furthermore, it has been shown that treated wastewater frequently contains triclosan, a chemical used in home and personal hygiene products as mouthwash, toothpaste, soaps, deodorants, and disinfecting lotions.^21^ Nanoparticles (NP) are another type of micropollutant found in wastewater effluents. Nanoparticles are found in trace numbers in wastewater, and eventually find their way into aquatic habitats due to their use in medicine and several household items.

### 2.2 Wastewater treatment methods

**Fig. 4:**
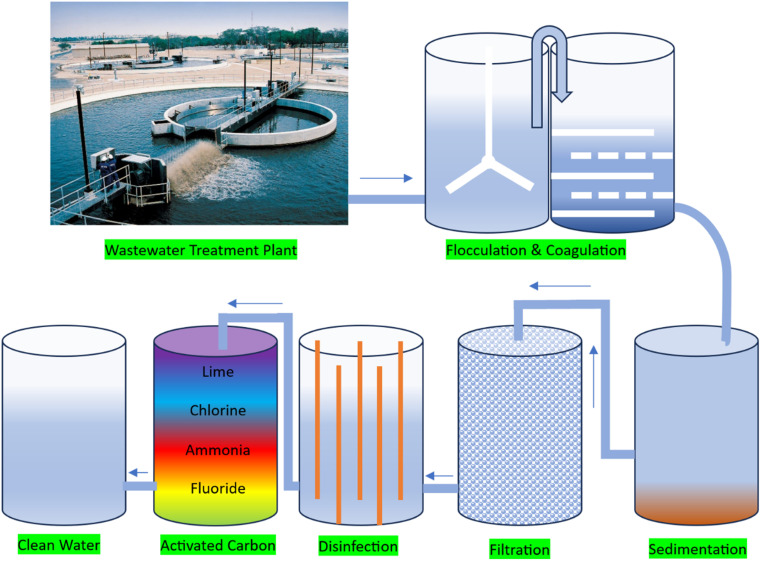
Stages in wastewater treatment plant.

Wastewater treatment is the process of removing contaminants from wastewater, making it safe to discharge into the environment or reuse. Presently, several wastewater treatment methods are used including physical treatment methods involve the removal of large particles and solids from wastewater through screening, sedimentation, and filtration.^22^ Biological treatment methods involve the use microorganisms to break down and remove organic contaminants from wastewater. The most common type of biological treatment is the use of activated sludge, which involves adding microorganisms to wastewater to digest organic matter and produce a sludge that can be removed and further treated.^23^ Chemical treatment methods involve the use of chemicals to remove contaminants from wastewater. The common chemical treatment processes include coagulation and flocculation, which involve adding chemicals to wastewater to cause solids to clump together and settle out of the water. Membrane filtration is a type of physical treatment method that uses ultra-filtration or reverse osmosis to remove contaminants from wastewater.^24^ This process is often used in conjunction with other treatment methods to further purify water. Disinfection is the process of killing or inactivating disease-causing microorganisms in wastewater. The most common disinfection method is the use of chlorine, which is added to water to kill bacteria and viruses. Each of these wastewater treatment methods has its own strengths and weaknesses, and the most effective approach will depend on the specific contaminants present in the wastewater and the desired level of treatment.^25^ Many treatment plants use a combination of these methods to ensure that the wastewater is thoroughly treated before it is discharged or reused. This type of treatment plant is shown in Fig. 4 with the stages followed in conventional wastewater treatment plants.

#### 2.2.1 Flocculation

Flocculation is a chemical treatment process that is used in wastewater treatment to remove suspended solids and other contaminants from the water. This process involves the addition of chemicals to wastewater, causing the suspended solids to clump together and form larger particles, called flocs, which can be more easily removed from the water. The chemicals used in flocculation are called flocculants, which are typically added to the wastewater after the primary treatment has been completed.^26^ The most common type of flocculant is aluminium sulfate, also known as alum, but other chemicals such as ferric chloride and polyacrylamide can also be used. The flocculation process is typically carried out in a large tank called a flocculator. The wastewater is slowly mixed with the flocculant, allowing the flocs to form and grow in size. As the flocs grow, they become heavier and sink to the bottom of the tank, where they can be removed using sedimentation or filtration. One advantage of the flocculation process is that it can be used to remove a wide range of contaminants from wastewater, including suspended solids, organic matter, and some metals.^27^ Flocculation is a relatively simple and inexpensive process compared to other treatment methods. However, the flocculation process has some limitations. For example, it may not be effective at removing very small particles or dissolved contaminants from the water. Additionally, the use of chemicals in the flocculation process can create additional waste, which requires proper disposal. Overall, the flocculation method is an important part of wastewater treatment and is often used in combination with other treatment methods to ensure that the water is thoroughly purified before it is discharged or reused.^28,29^

#### 2.2.2 Chemical precipitation

Chemical precipitation is a commonly used wastewater treatment method, which involves the use of chemicals to remove contaminants from wastewater. This method is based on the principle of forming insoluble solid particles that can be removed through sedimentation or filtration. In chemical precipitation, chemicals such as lime, ferric chloride, and aluminium sulfate are added to wastewater to form solid particles that can be easily removed. The chemicals react with the contaminants in the water to form a precipitate, which settles to the bottom of a sedimentation tank or is removed through filtration. One of the primary benefits of chemical precipitation is that it is effective in removing a wide range of contaminants, including heavy metals, phosphorus, and suspended solids. It is also relatively simple to implement and requires minimal equipment. This method involves the addition of chemicals such as aluminium sulphate and polyelectrolytes to wastewater to form solid particles, which can be removed through sedimentation. This method is used to remove hardness-causing ions such as calcium and magnesium from wastewater. Chemicals such as lime or soda ash are added to the water to precipitate these ions, which can be removed through sedimentation or filtration. Chemical precipitation is often used to remove phosphorus from wastewater, which can contribute to the growth of harmful algal blooms. Chemicals such as ferric chloride and aluminium sulphate are added to water to precipitate phosphorus, which can be removed through sedimentation or filtration.^30^ Overall, chemical precipitation is a widely used and effective wastewater treatment method that can remove a variety of contaminants. However, it has some limitations, including the production of large amounts of sludge, which must be disposed properly, and the potential for chemical overdosing, which can lead to environmental problems.

#### 2.2.3 Activated charcoal

Activated charcoal, also known as activated carbon, is a highly porous material that is commonly used in wastewater treatment to remove contaminants. Activated charcoal treatment method involves passing wastewater through a bed of activated charcoal, which adsorbs contaminants on its surface and removes them from water. The process of activated charcoal treatment begins with the preparation of the activated charcoal. The charcoal is typically made from organic materials such as coconut shells, wood, and coal, which are heated to high temperatures in the absence of oxygen to create a highly porous material with a large surface area. This surface area is key to the effectiveness of activated charcoal in wastewater treatment, given that it provides a large area for the adsorption of contaminants. Once the activated charcoal is prepared, it is typically placed in a tank or filter bed through which the wastewater flows.^31^ As the water passes through the charcoal bed, contaminants such as organic compounds, pesticides, and pharmaceuticals adsorb on to the charcoal surface and are removed from the water. The adsorption process is highly effective, given that the large surface area of the charcoal provides ample opportunity for the contaminants to come in to contact with the adsorbent surface. Activated charcoal treatment is highly effective for the removal of a wide range of contaminants from wastewater, including volatile organic compounds (VOCs), synthetic dyes, and heavy metals. It is also effective for removing organic matter from water, which can help reduce the amount of sludge produced during subsequent treatment processes. One of the major advantages of activated charcoal treatment is that it is a passive process, and thus does not require the use of chemicals or energy. This makes it a relatively low-cost treatment option, particularly for small-scale wastewater treatment systems. However, the effectiveness of the treatment may be limited by the capacity of the charcoal bed, and the charcoal may need to be replaced periodically to maintain its effectiveness.^32^ Overall, the use of activated charcoal is a highly effective wastewater treatment method to remove a wide range of contaminants from water. Its effectiveness, low cost, and simplicity make it a popular treatment option for both large-scale and small-scale wastewater treatment systems.

#### 2.2.4 Reverse osmosis

Reverse osmosis (RO) is a type of membrane filtration used in wastewater treatment to remove contaminants from water. It involves the use of a semi-permeable membrane, which allows water molecules to pass through, while blocking the passage of dissolved solids, organic compounds, and other contaminants.^33^ RO is an effective method for removing a wide range of contaminants from wastewater, including salts, minerals, bacteria, viruses, and organic compounds. In the RO process, wastewater is forced through a series of membranes under high pressure, which causes the water molecules to pass through the membrane, while contaminants are left behind. The membranes used in RO are very fine and have a pore size of 0.0001 μm or less, making them capable of removing even very small particles from the water. The RO process typically includes several stages of treatment, including pre-treatment, membrane filtration, and post-treatment.^34^ Pre-treatment involves the removal of large particles and solids from the wastewater through screening and sedimentation, as well as the adjustment of pH and other chemical parameters to ensure that the water is compatible with the RO membrane. During the membrane filtration stage, the wastewater is passed through the RO membrane under high pressure, which removes dissolved solids, organic compounds, and other contaminants from the water. Subsequently, the permeate or treated water is collected and stored for further treatment or discharge. Post-treatment may involve the addition of chemicals to the permeate to adjust its pH or to disinfect the water, depending on the desired end use.^35^ RO is often used in conjunction with other treatment methods, such as activated sludge and chemical treatment, to provide a comprehensive wastewater treatment solution. One of the main advantages of RO is its ability to remove a wide range of contaminants from wastewater, making it a versatile treatment method for a variety of applications. However, the high energy requirements and maintenance costs associated with their process can make it more expensive than other treatment methods in some cases. Nonetheless, RO is an important tool in the wastewater treatment toolbox and is used in a variety of industrial, municipal, and agricultural applications globally.^36^

#### 2.2.5 Ultraviolet disinfection

Ultraviolet (UV) disinfection is a common method used in wastewater treatment to remove harmful microorganisms from water. This process involves exposing wastewater to UV light, which disrupts the DNA of microorganisms, preventing their reproduction. The UV disinfection process involves passing wastewater through a chamber that contains UV lamps.^37^ The lamps emit high-intensity UV light, typically at a wavelength of 254 nanometres, which is highly effective at killing bacteria, viruses, and other harmful microorganisms. The intensity and duration of the UV light exposure are carefully controlled to ensure that microorganisms are effectively destroyed, while minimizing the energy required for the process. One of the advantages of UV disinfection is that it does not introduce any chemicals into the water, which can be beneficial for applications where the water will be reused or discharged into the environment.^38^ Additionally, UV disinfection is highly effective at removing a wide range of microorganisms, including those are resistant to chemical disinfection methods. However, there are also some limitations to UV disinfection. For example, the process is only effective at removing microorganisms and does not remove other contaminants such as chemicals or solids. Additionally, the effectiveness of UV disinfection can be affected by the quality of the water being treated, such as the level of turbidity or the presence of certain organic compounds. Overall, UV disinfection is a valuable tool for wastewater treatment facilities looking to remove harmful microorganisms from the water.^39^ It is a highly effective and environmentally-friendly method that can be used in conjunction with other treatment methods to provide a comprehensive solution for treating wastewater.

#### 2.2.6 Ultra-filtration

Ultra-filtration is a type of membrane filtration that is used in wastewater treatment to remove suspended solids, bacteria, and other contaminants from the water. This process involves forcing wastewater through a membrane with pores that are too small for contaminants to pass through, while allowing the water to pass through freely. The result is purified water that can be reused or discharged into the environment. The ultra-filtration process typically involves several steps. Firstly, the wastewater is screened to remove large solids and particles. Then, it is pumped through a series of ultra-filtration membranes, which remove smaller particles, bacteria, and other contaminants.^40^ The membranes are made of a variety of materials, including polymeric materials, ceramic, or metallic materials, and can be configured in various shapes and sizes depending on the specific application. One of the main advantages of ultra-filtration is that it is a highly efficient process that can remove a wide range of contaminants from wastewaters.^41^ Also, it can be used in conjunction with other treatment methods, such as reverse osmosis and biological treatment, to further purify water. Additionally, ultra-filtration is a relatively simple process that can be easily automated and controlled. However, there are also some challenges associated with ultra-filtration. One of the main challenges is the fouling of the membrane, which occurs when contaminants build up on its surface, reducing its effectiveness. This can be addressed through regular maintenance and cleaning of the membranes, as well as the use of specialized cleaning chemicals. Another challenge is the high capital and operating costs associated with ultra-filtration systems. The membranes used in the process can be expensive, and the energy required to pump water through the membranes can be significant. However, as technology improves and more efficient systems are developed, the cost of ultra-filtration is expected to decrease.^42^ Overall, ultra-filtration is an effective method for wastewater treatment that can be used to produce high-quality purified water for reuse or discharge into the environment. Although there are some challenges associated with this process, they can be addressed through careful planning, maintenance, and optimization of the treatment system.

#### 2.2.7 Electro-coagulation

Electro-coagulation is a waste-water treatment method that uses an electric current to remove contaminants from water. It is a type of chemical treatment that involves the destabilization of contaminants through the creation of charged ions. It is possible to employ a variety of electrode materials, including aluminium, stainless steel, and iron.^43^ This method causes the metal ions to dissolve in the water and create charged particles. Subsequently, the charged particles attract and coagulate the contaminants in the water, causing them to settle out or float to the surface, where they can be easily removed. One of the advantages of electro-coagulation is that it can remove a wide range of contaminants from wastewater, including suspended solids, organic matter, and heavy metals.^44^ Also, it is effective at removing contaminants that are difficult to treat with other methods, such as oils and emulsions. Compared to bigger facilities, small localized treatment facilities for small-scale electro-coagulation can be more economical and require less energy, making them suitable for operation using renewable energy sources. Furthermore, the chemical consumption cost is zero given that no extra chemicals are employed to increase the electrical conductivity or modify the pH.^45^ However, electro-coagulation also has some limitations. This method requires a source of electricity, which can increase the operating costs of the system. Also, it can produce sludge, which must be properly disposed to prevent environmental harm. However, despite these limitations, electro-coagulation has shown promise as a wastewater treatment method in a variety of applications, including industrial wastewater treatment, municipal waste water treatment, and the treatment of contaminated groundwater.^46^ As research and development continue, electro-coagulation may become an increasingly important tool in the effort to provide safe and sustainable water resources for communities around the world.

#### 2.2.8 Ion exchange

Ion exchange is a wastewater treatment method that involves removing unwanted ions from the water by exchanging them with other ions of similar charge. This process involves passing the wastewater through a resin bed that is filled with an ion exchange material, which is typically composed of synthetic or natural polymers. During ion exchange, the resin beads attract and hold on to specific ions, depending on their charge and size. For example, anion exchange resins will attract and hold on to negatively charged ions such as nitrates and sulfates, while cation exchange resins will attract and hold onto positively charged ions such as calcium and magnesium.^47^ The ion exchange process can be used to treat a variety of wastewater types, including industrial and domestic wastewater. It is particularly effective for treating wastewater that contains high levels of dissolved salts or heavy metals, such as those found in industrial effluent. One of the advantages of ion exchange is that it can be tailored to selectively remove specific contaminants from the wastewater. This means that the ion exchange resin can be designed to target particular ions or chemicals that need to be removed, while leaving others in the water. This makes the process highly versatile and adaptable to different types of wastewaters. However, there are also some limitations to ion exchange. One of the main drawbacks is that the resin bed can become exhausted over time, meaning that it can no longer effectively exchange ions and needs to be replaced or regenerated. Regeneration involves flushing the resin bed with a chemical solution that removes the unwanted ions and replaces them with fresh ones, allowing the resin to be reused.^48^ Ion exchange is an effective and flexible wastewater treatment method that can be used to remove a wide range of contaminants from wastewater. However, it requires careful monitoring and maintenance to ensure that the resin bed is functioning effectively and to avoid overloading the system. Details of the principle of operation of the different treatment methods together with their merits and demerits are discussed in Table 1.

**Table 1:** Treatment methods together with their merits and demerits

Method	Principle operation	Advantages	Disadvantages
Flocculation	Aggregates suspended particles using a coagulating agent	Low cost, easy to operate, effective at removing larger particles	Not effective for smaller particles, can create large amounts of sludge
Chemical precipitation	Converts dissolved pollutants into solid particles using a chemical reagent	Effective at removing dissolved pollutants, low cost, easy to operate	Generates large amounts of sludge, requires careful handling of chemical reagents
Activated charcoal	Absorbs pollutants onto its surface	Effective at removing organic pollutants, can be reused, low cost	Requires frequent replacement, less effective at removing inorganic pollutants
Reverse osmosis	Uses a semi-permeable membrane to remove dissolved pollutants	Effective at removing dissolved pollutants, can be used for drinking water	Requires high-pressure, high-energy consumption, produces wastewater
Ultra-filtration	Uses membrane to remove suspended particles	Effective at removing suspended particles, low energy consumption	Membrane can be clogged easily, not effective at removing dissolved pollutants
Electro coagulation	Uses an electric field to destabilize and remove pollutants	Effective at removing suspended particles, can be used for a wide range of pollutants	High energy consumption and requires frequent maintenance
Ion exchange	Removes dissolved pollutants by exchanging ions with a resin	Effective at removing dissolved pollutants, can be used for a wide range of pollutants	Requires frequent resin replacement, not effective at removing larger particles

## 3 Algae-based pollutant removal strategies

**Fig. 5:**
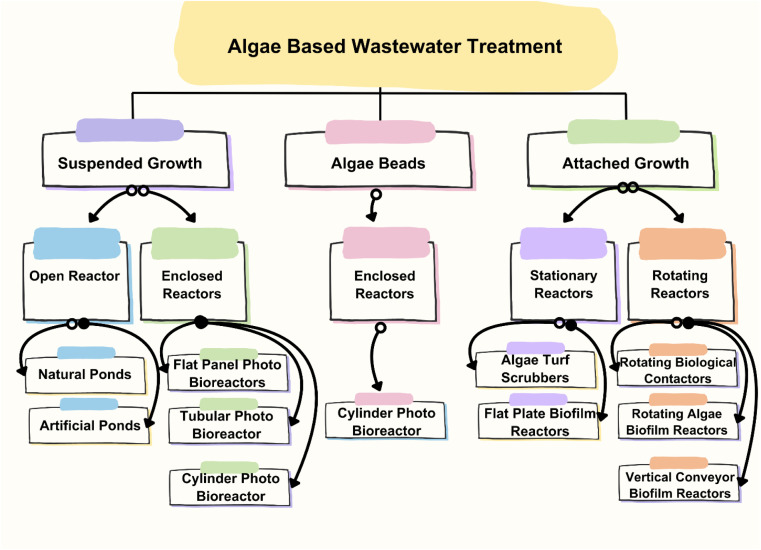
Algae-based wastewater treatment strategies.

Algae-based pollutant removal strategies, as shown in Fig. 5, involve the use of algae to remove pollutants from various sources, including wastewater, agricultural runoff, and industrial effluents. Algae-based treatment technologies have gained popularity due to their low cost, environmental friendliness, and ability to remove a wide range of pollutants.^49^ One of the primary methods for algae-based pollutants removal is through the use of algae ponds. Algae ponds are shallow, artificial ponds that contain algae. The algae in these ponds are exposed to sunlight, which promotes photosynthesis. During photosynthesis, the algae absorb nutrients such as nitrogen and phosphorus, which are often the main pollutants in wastewater and agricultural runoff. Consequently, algae ponds can effectively remove these pollutants from waters.^50^ Another method for algae-based pollutants removal is through the use of algae bioreactors. Algae bioreactors are closed systems that are designed to cultivate algae. These bioreactors can treat wastewater, industrial effluents, and other sources of pollutants. In these bioreactors, algae are grown under controlled conditions, where their growth and the pollutant removal rate can be optimized.

**Fig. 6:**
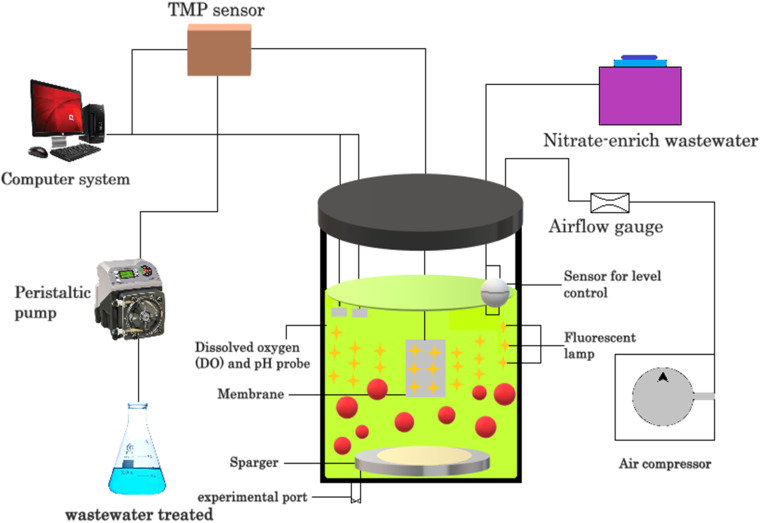
Pollutant removal through algae membrane reactors.

Algae-based pollutant removal through the use of algae-based membranes is depicted in Fig. 6. These membranes are made up of algae and can effectively remove pollutants from water through a combination of physical and biological processes. The algae in the membrane absorb pollutants, while the membrane itself acts as a filter to remove them from the water. Another emerging method for algae-based pollutant removal is through the use of genetically engineered algae. These algae are modified to enhance their ability to remove pollutants from water.^51^ For example, scientists have developed algae that can absorb heavy metals such as cadmium and lead, which are common pollutants in industrial effluents. The algae produced in algae ponds and bioreactors can be used as a source of biofuel or fertilizer. Additionally, the use of algae-based pollutant removal strategies can help to reduce greenhouse gas emissions by capturing carbon dioxide during photosynthesis. Algae-based pollutant removal strategies are a promising and effective solution to the problem of water pollution. These methods have the potential to improve the quality of water, while also providing other benefits, such as the production of biofuels and fertilizers. With continued research and development, algae-based pollutant removal strategies will play a significant role in preserving natural resources.^52^

### 3.1 Physical arrangement of an algae membrane reactor

A membrane reactor is a type of chemical reactor that utilizes a membrane to separate the reactants and products of a chemical reaction. This membrane provides a physical barrier, which allows the selective transport of certain molecules or ions, allowing controlled reactions that are not possible with traditional reactors. The physical arrangement of a membrane reactor can vary depending on the specific application and type of membrane used. Generally, a membrane reactor consists of a reactor vessel, a membrane module, and a feed and product system. The reactor vessel contains the catalyst and the reactants, which are typically fed in to the reactor continuously or intermittently.^53^ The membrane module is installed within the reactor vessel and separates the reactants from the products. The feed and product system is responsible for delivering the reactants to the reactor vessel and collecting the products from the membrane module. The membrane module in a membrane reactor is designed to have a high surface area and good permeability to allow the efficient separation of the reactants and products. It can be constructed using a variety of materials, including ceramics, polymers, and metals. The membrane can be flat or tubular and can be arranged in parallel or in series depending on the desired reaction conditions. The membrane can also be coated with a catalyst to enhance the reaction rate or selectivity. One advantage of using a membrane reactor is that it can overcome thermodynamic limitations and enable reactions that are not possible in traditional reactors. For example, membrane reactors can allow the removal of reaction products from the reaction mixture, which can shift the equilibrium towards the desired product. Additionally, membrane reactors can enable reactions that require high temperatures or pressures by separating the reactants from the reaction products and preventing unwanted side reactions. Membrane reactors have numerous applications in the chemical, petrochemical, and pharmaceutical industries. They are commonly used in processes such as hydrogenation, dehydrogenation, and oxidation reactions.^54^ Membrane reactors can also be used in the production of fine chemicals and synthesis of polymers. In conclusion, the physical arrangement of a membrane reactor consists of a reactor vessel, a membrane module, and a feed and product system. The membrane module separates the reactants from the products and can be constructed using a variety of materials and designs. Algae membrane reactors have several advantages compared to traditional reactors, including the ability to overcome thermodynamic limitations and enable new reactions. They have numerous applications in various industries, making them promising technology for the future.

### 3.2 Types of arrangements in algae-based pollutant removal

Algal bioremediation systems offer several advantages compared to other technologies for the removal of emerging pollutants. These systems harness the natural capabilities of algae to degrade or absorb various types of pollutants, providing a cost-effective and environmentally friendly approach for remediation. In comparison to conventional methods, algae-based technology has been proposed as a potential treatment for reducing BOD, removing N and/or P, inhibiting coli forms, and removing heavy metals from wastewater. Algal biomass can also be used for methane generation, composting, liquid fuel (pseudo vegetable fuel) production, animal feed or aquaculture and fine chemical manufacture. Algae-based pollutant removal strategies are becoming increasingly popular due to the high efficiency of algae in removing pollutants from wastewater. There are several types of arrangements used in these strategies, each with its own advantages and disadvantages. In this section, some of the most common types of arrangements used in algae-based pollutant removal strategies are discussed.

**Fig. 7:**
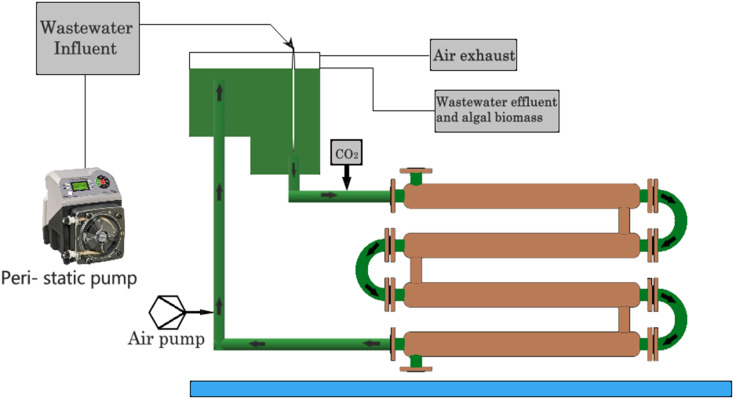
Lab-scale closed photobioreactor for pollutant removal.

Open pond systems are the most common type of algae-based pollutant removal system. In this arrangement, wastewater is pumped into open ponds or raceways, where algae grow and remove pollutants from the water. The ponds can be shallow or deep and can be made from various materials such as plastic, concrete, and earth. One of the biggest advantages of open pond systems is their low cost, making them accessible to many communities. Additionally, open pond systems require low energy inputs and can be used to produce biofuels or other value-added products. However, open pond systems also have several disadvantages.^55^ For example, they are sensitive to changes in weather conditions and can be impacted by environmental factors such as temperature, sunlight, and rainfall. Moreover, open pond systems require a large amount of land area, which can be a limiting factor in densely populated areas. Additionally, open pond systems may be prone to contamination from outside sources, which can compromise the quality of the water. Closed photo bioreactors are another type of algae-based pollutant removal system. In this arrangement, algae are grown in closed vessels that are exposed to light. The vessels can be made from various materials such as glass and plastic, and can be designed to control the temperature, light, and nutrient inputs. One such system is shown in Fig. 7.

One of the biggest advantages of closed photo bioreactors is their high efficiency, which allows a smaller footprint and higher production of algae biomass. Additionally, closed photo bioreactors can be used to control the environmental conditions, which can result in higher-quality algae biomass. However, closed photo bioreactors also have several disadvantages. For example, they can be expensive to construct and maintain, making them less accessible to many communities.^56^ Additionally, closed photo bioreactors require a constant supply of energy to maintain the environmental conditions, which can increase the cost of the operation. Finally, closed photo bioreactors may be prone to bio film formation or contamination, which can affect the quality of the algae biomass.

Hybrid systems are a combination of open pond systems and closed photo bioreactors. In this arrangement, wastewater is first treated in an open pond system, and then transferred to a closed photo bioreactor for further treatment and harvesting of the algae biomass. One of the biggest advantages of hybrid systems is their flexibility, given that they can be designed to maximize the advantages of both open pond systems and closed photo bioreactors. Additionally, hybrid systems can be used to produce a wide range of algae-based products, including biofuels, food supplements, and pharmaceuticals. However, hybrid systems also have several disadvantages. For example, they are complex to design and operate, requiring expertise in both open pond systems and closed photo bioreactors. Additionally, hybrid systems require a higher level of maintenance than individual open pond systems and closed photo bioreactors. Finally, hybrid systems may be more expensive to construct and operate than either open pond systems or closed photo bioreactors alone.^57^ Overall, open pond systems are the most common and cost-effective type of system, while closed photo bioreactors are the most efficient and produce higher-quality algae biomass. Hybrid systems combine the advantages of both open pond systems and closed photo bioreactors but are also more complex and expensive to design and operate.

## 4 Physicochemical factors influencing algal-bioremediation for emerging pollutant removal

Algal bioremediation has been gaining popularity as a potential solution for emerging pollutant removal in water bodies. However, the effectiveness of this technique depends on several physicochemical factors. The characteristics of emerging pollutants, such as their solubility, volatility, and persistence, can influence their uptake by algae. Some pollutants may be more easily absorbed by algae than others, and the efficiency of algal bioremediation can be affected by the properties of the pollutants. Different algal species have varying abilities to uptake and metabolize emerging pollutants.^58^ Therefore, the selection of appropriate algal species is crucial for efficient algal bioremediation. Maintaining the optimal pH, temperature, light, and nutrient concentrations, and selecting appropriate algal species can help maximize the efficiency of this technique. Additionally, understanding the characteristics of the pollutants is essential to ensure their effective uptake and removal by the algae. The average values for the physico-chemical properties are presented in Table 2.

**Table 2:** Values for physico-chemical properties of algae bio-remediation

Parameter	Value ranges
Light intensity	55–145 μmol m^−2^ s^−1^
pH	7.8–8.4
Temperature	21–260 °C
Nutrient concentration	1–6 mg L^−1^
Pollutant concentration	1–11 mg L^−1^
Dissolved oxygen levels	>6 mg L^−1^
Algal biomass density	0.6–1.1 g L^−1^
Residence time	4–8 days
Mixing/agitation	25–45 rpm
Carbon dioxide supply	1–6% v/v
Nutrient-removal efficiency	81–96%

### 4.1 pH

The pH of water is an important physicochemical factor that can influence algal bioremediation for emerging pollutant removal. Algae have specific pH ranges that are optimal for their growth and activity, and changes in pH outside this range can significantly impact their efficiency in removing emerging pollutants from water. Most algae prefer a pH range of 6.5 to 9, with some species able to tolerate a broader range. If the pH is outside this optimal range, it can affect the solubility and bioavailability of emerging pollutants, making them less accessible for uptake by algae. Additionally, extreme pH values can cause cellular damage or death, which can lead to reduced algal growth and activity, and ultimately lower rates of pollutant removal. The effect of pH on algal bioremediation is dependent on the chemical nature of the emerging pollutant.^59^ Some pollutants may be more readily absorbed at a particular pH, while others may require a different pH range for efficient uptake. For example, some organic pollutants are more easily absorbed by algae at a neutral pH, while others are better absorbed under acidic or alkaline conditions. Thus, controlling the pH levels can help to optimize algal bioremediation for emerging pollutant removal. If the pH is too low, adding alkaline substances, such as sodium hydroxide and calcium carbonate, can help increase the pH to a more optimal level. Conversely, if the pH is too high, adding acidic substances, such as sulphuric acid and hydrochloric acid, can help lower the pH to the optimal level. However, care must be taken to ensure that the pH is not adjusted to extremes, which may harm the algae or other aquatic organisms. Maintaining an optimal pH range can help maximize the uptake of emerging pollutants by algae, while also promoting algal growth and activity. Therefore, it is important to consider pH as a key factor when designing and implementing algal bioremediation strategies for emerging pollutant removal.

### 4.2 Redox

Redox potential is another physicochemical factor that can influence algal bioremediation for emerging pollutant removal. The redox potential is a measure of the tendency of a system to acquire or lose electrons, which can impact the bioavailability and biodegradability of emerging pollutants. The redox potential plays a crucial role in oxidation–reduction reactions, which are important in the degradation of emerging pollutants. Some emerging pollutants, such as pharmaceuticals and personal care products, can be difficult to biodegrade because they are highly oxidized.^60^ However, algal bioremediation can facilitate the reduction of these pollutants, making them more biodegradable. Algae require electron acceptors, such as oxygen, to carry out metabolic processes. The redox potential can affect the availability of electron acceptors, which can impact the rate of algal bioremediation. Under anoxic conditions, for example, the lack of oxygen can slow down algal growth and reduce the efficiency of emerging pollutant removal. The redox potential can also impact the interactions between emerging pollutants and co-contaminants. In some cases, co-contaminants can interfere with the uptake and degradation of emerging pollutants by algae. The interactions between the chemical reagents and target pollutants determine how well the inhomogeneous processes remove the developing pollutants. Municipal agriculture, the rubber industry, textile industry, and other industrial streams are some of the wastewater streams in which microalgae are utilized for wastewater treatment. According to reports, *Chlorella vulgaris* has been used to treat wastewater that is discharged from the rubber latex industry. It reduces the total nitrogen and COD concentration by around 80% and 90%, respectively. In recent years, there have been many instances of the use of micro algal culture to remove heavy metals from wastewater. For example, *Chlorella vulgaris* has a high uptake of cadmium(ii). Comparably, it has been observed that *Spirulina platensis* consumes around 45% iron, 70% arsenic, 80% chromium, 85% manganese, 60% nickel, 50% copper, and 55% zinc. In ten days, ammonium and phosphates were extracted from synthetic wastewater using *Chlorella vulgaris*. *Desmodesmus* sp., *Oscillatoria*, and *Arthrospira* were used to treat effluent from a lagoon plant using an open pond reactor, removing 83% and 60% of nitrogen and phosphorus in 19 days, respectively. 100% of the phosphate and 80% of the wastewater were eliminated by *Spirulina* sp. grown utilizing aquaculture effluent on an outdoor trial scale.^61^ The redox potential can also affect the bioavailability of emerging pollutants to algae. In some cases, emerging pollutants may be highly oxidized, and therefore not easily bioavailable to algae. However, the reduction of these pollutants through oxidation–reduction reactions can make them more bioavailable, leading to increased uptake and removal by the algae. The redox potential is an important physicochemical factor that can influence the efficiency of algal bioremediation for emerging pollutant removal. Understanding the role of the redox potential in oxidation–reduction reactions, electron acceptors, co-contaminants, and bioavailability can help optimize the conditions for algal bioremediation and enhance the removal of emerging pollutants from water bodies.

### 4.3 Temperature

Temperature is a crucial physicochemical factor that can greatly influence the efficiency of algal bioremediation for emerging pollutant removal. Algae have specific temperature requirements for their growth and activity, where variations in temperature can have significant effects on their ability to remove pollutants from water bodies. Temperature can influence the algal growth rates and biomass production, which affects the uptake and removal of emerging pollutants. For example, high temperatures can accelerate algal growth and increase the rate of pollutant uptake, but also lead to algal die-off if the temperature exceeds the optimal range for a particular species.^62^ Conversely, low temperatures can slow down algal growth and reduce their ability to remove pollutants from water. The optimal temperature range for algal bioremediation varies depending on the species of algae used and the specific pollutants being targeted. Generally, algal species used for bioremediation prefer temperatures ranging from 15 °C to 30 °C, although some species can tolerate temperatures as low as 5 °C or as high as 35 °C. It is important to maintain temperatures within the optimal range to maximize the efficiency of algal bioremediation. Temperature can also affect the physical and chemical properties of the water, which can influence the solubility and bioavailability of emerging pollutants. For example, temperature can affect the pH and dissolved oxygen concentration of the water, which can impact the uptake and removal of pollutants by algae. Temperature is an important physicochemical factor that can significantly impact the efficiency of algal bioremediation for emerging pollutant removal. Maintaining the temperature in the optimal range for algal growth and activity can help maximize the rate of pollutant uptake and removal by algae. It is important to consider the specific algal species being used and the characteristics of the emerging pollutants to determine the optimal temperature range for effective bioremediation.

### 4.4 Duration and intensity of light exposure

The duration and intensity of light exposure are important physicochemical factors that can influence algal bioremediation for emerging pollutant removal. Light is essential for algal growth and activity given that it provides the energy required for photosynthesis. In the absence of light, the growth and activity of algae are significantly reduced or even halted. Also, the intensity of light exposure can affect the rate of uptake of emerging pollutants by algae. Higher light intensities can result in the faster uptake and removal of pollutants. However, excessively high light intensities can cause photo-inhibition and result in the production of reactive oxygen species, which can damage the algal cells and reduce their efficiency. Therefore, it is important to maintain the optimal light intensity levels for efficient algal bioremediation. The duration of light exposure also plays a role in algal bioremediation. Algae require a certain amount of light exposure to maintain their growth and activity.^63^ However, extended periods of light exposure can result in photo-inhibition and reduce the efficiency of algal bioremediation. Algal growth and activity are typically the highest during the light period of the day and reduced or halted during the dark period. Therefore, the duration of light exposure should be optimized to ensure efficient algal bioremediation. The specific duration and intensity of light exposure required for efficient algal bioremediation can vary based on the species of algae and the pollutants being targeted. In some cases, a combination of light and dark periods may be required to promote the optimal algal growth and activity. For example, some studies have shown that a light/dark cycle of 12 h light/12 h dark can optimize the efficiency of algal bioremediation for certain pollutants. The duration and intensity of light exposure are important physicochemical factors that can influence the efficiency of algal bioremediation for emerging pollutant removal. Thus, maintaining the optimal light intensity and duration levels can help maximize algal growth and activity, while avoiding photoinhibition. However, the specific light requirements may vary depending on the algae species and the pollutants being targeted.

### 4.5 Hydraulic retention time, adsorbent size and concentration of emerging pollutants

One strategy for removing EPs from water sources is the use of adsorbents. Adsorbents are materials that can selectively remove pollutants from water by binding them to their surface. The performance of an adsorbent is influenced by several factors, including hydraulic retention time, adsorbent size, and concentration of EPs. The hydraulic retention time (HRT) is the time that water spends in a treatment system, which has a significant impact on the performance of an adsorbent. A longer HRT allows a longer contact time between the adsorbent and the water, increasing the likelihood of adsorption. However, a longer HRT also increases the cost of the treatment system, given that more time and resources are required to treat the water. Therefore, the HRT should be optimized to balance the cost of treatment with the desired level of pollutant removal. The size of the adsorbent particles is another important factor that affects their performance. Smaller particles provide a greater surface area for adsorption, which can improve the adsorption capacity of the material. However, smaller particles can also lead to problems such as channelling and clogging in the treatment system, which can reduce the overall efficiency of the system.^64^ Therefore, the size of the adsorbent particles should be optimized based on the specific application and the characteristics of the EPs being treated. The concentration of EPs in the water is another factor that affects the performance of an adsorbent. Higher concentrations of EPs can lead to saturation of the adsorbent and a decrease in its ability to remove pollutants. Therefore, it is important to determine the optimal concentration of EPs for a given adsorbent and ensure that the treatment system is designed to handle the expected concentration of pollutants. The hydraulic retention time, adsorbent size, and concentration of EPs should be carefully considered when designing and optimizing treatment systems to ensure the effective removal of pollutants, while minimizing costs and maximizing the efficiency.

## 5 Technical analysis of algal-bioremediation systems for the removal of emerging pollutants

It is both ecologically and technologically possible to use micro-algae as a substitute biological wastewater treatment solution. Also, it is economically competitive considering that traditional systems require installation fees in addition to the enormous expenses related to growing a micro-algae plant, which are the subject of this discussion. Micro-algae systems have negligible or no operating expenses, which makes them overall much more sustainable than traditional systems. Under static culture circumstances, it may not be feasible to employ a micro-algal-bacterial consortium with strong settling qualities because maintaining the cells in a suspension negatively affects how well the consortium treats wastewater. Because intermittent aeration uses less energy than continuous aeration, it is recommended for static systems. Therefore, more research and development should focus on a reactor architecture that alternates between intermittent aeration and CO_2_ injection. Several by-product streams from the food business, such as molasses streams, dairy industry by-product streams, and fruit processing sector industrial by-product streams, have high saccharide concentrations, which can be investigated in this regard. Unlike some other remediation technologies that are specific to certain pollutants, algal systems have broader applicability.^65^ Algae possess high metabolic rates and can efficiently degrade or absorb pollutants. They produce enzymes and other compounds that can break down complex organic molecules, converting them into simpler and less harmful forms. Algal bioremediation systems have shown impressive removal efficiencies for different pollutants, effectively reducing their concentrations in contaminated water bodies. Algae require nutrients such as nitrogen and phosphorus for growth. Thus, in polluted water bodies, excess nutrients often contribute to eutrophication and harmful algal blooms. Algal bioremediation systems can help address this issue by utilizing the excess nutrients as a resource for algal growth. The algae absorb the nutrients, reducing their availability for other organisms, and thus mitigating eutrophication.^66^ Algae are photosynthetic organisms that utilize sunlight as an energy source for growth. This unique characteristic enables algal bioremediation systems to operate using solar energy, making them highly sustainable and cost-effective compared to energy-intensive technologies. The sunlight-driven growth of algae not only facilitates the removal of pollutants but also contributes to the overall health of aquatic ecosystems by increasing oxygen production. The biomass generated during algal bioremediation can have additional benefits beyond pollutant removal. Algal biomass can be harvested and used as a feedstock for the production of biofuels, bioplastics, and other value-added products. This concept of integrated bioremediation and biomass valorisation offers potential for economic incentives and creates a circular economy model. Algal bioremediation is a non-toxic approach for pollutant removal. Unlike some chemical or physical remediation methods, algal systems do not introduce additional harmful substances into the environment.^67^ Algae naturally metabolize and transform pollutants into less toxic forms or incorporate them into their biomass, thereby reducing the ecological risks associated with the pollutants. Also, algal bioremediation systems can be implemented on various scales, ranging from small-scale applications in laboratory settings to large-scale installations in industrial or municipal wastewater treatment plants. They can be tailored to specific site conditions and pollutant types, offering flexibility in design and application. This scalability makes algal bioremediation a viable option for addressing emerging pollutants in different contexts. Algal bioremediation systems provide numerous advantages for the removal of emerging pollutants.^68^ Their versatility, high removal efficiency, nutrient recycling capabilities, solar energy utilization, biomass valorisation potential, non-toxic nature, and scalability make them an attractive and sustainable alternative to other remediation technologies. By harnessing the power of nature, algal bioremediation systems offer a promising solution for tackling emerging pollutants and promoting environmental health. A summary of the merits and demerits of algal bioremediation systems compared with other technologies is listed in Table 3.

**Table 3:** Merits and demerits of algae bioremediation technology

Algal bioremediation systems	Other technologies
With 80–100% removal efficiency, algae species such as *Chlorella vulgaris*, *Chlorella pyrenoidosa*, and *Chlorella minutissima* can remove pollutants including NO_3_, NH_3_, and PO_4_. For the elimination of NH_3_ and PO_4_, *Mucidosphaerium pulchellum* can achieve a removal efficiency of more than 60%. An algal bacterial consortium can remove PO_4_ and NO_3_ pollutants with a 90% removal rate. *Scenedesmus dimorphus* is an algae species that can remove heavy metals such as Cu(ii) with an efficiency close to 76%	Limited removal efficiency for certain emerging pollutants requiring additional treatment steps
Cost-effective and environmentally friendly approach, utilizing natural processes and renewable resources	Often expensive and energy-intensive, requiring advanced equipment and chemical treatments
Algae have a high growth rate and biomass productivity, enabling rapid pollutant uptake and degradation	Slower degradation rates in other technologies, leading to longer treatment times
Algal bioremediation can be implemented in both wastewater treatment plants and natural water bodies, offering versatility	Limited applicability to specific treatment settings, restricting their use in certain environments
Algae can remove pollutants through various mechanisms, including adsorption, bioaccumulation, and enzymatic degradation	Reliance on a single mechanism in other technologies, which may not be as efficient for certain pollutants
Algal bioremediation systems can be easily scaled up or down based on the volume of water to be treated	Limited scalability of other technologies, making them less suitable for large-scale applications
Algae have the potential for the simultaneous removal of multiple pollutants, allowing integrated treatment approaches	Often designed for the removal of specific pollutants, requiring separate treatment systems for different contaminants
Algal bioremediation can enhance the water quality by reducing nutrient uptake by algae	Lack of nutrient removal capabilities in other technologies, leading to potential eutrophication issues
Algae biomass generated during the treatment process can be harvested and used for biofuel production or as a valuable resource	Limited value-added opportunities for by-products generated by other treatment methods
Algal bioremediation systems offer the potential for long-term sustainability and ecological restoration of polluted water bodies	Limited ecological benefits and potential for sustainable restoration in other technologies

## 6 Challenges involved in the removal of emerging pollutants using algal-bioremediation systems

**Fig. 8:**
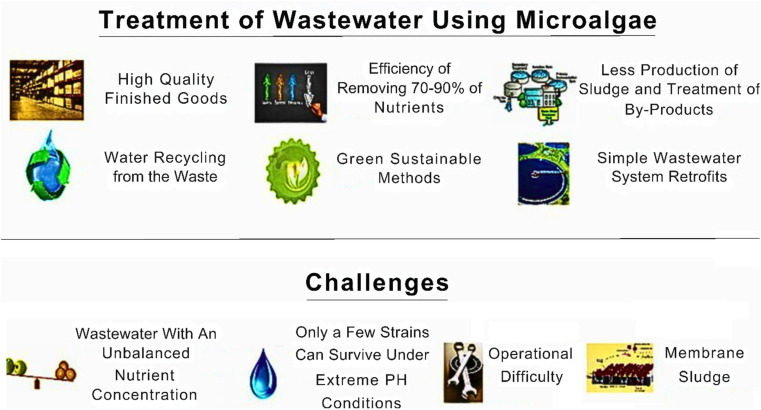
Technical advantages and challenges of using algae-based bioremediation systems.

Algal bioremediation systems have gained attention as a potential solution for the removal of emerging pollutants from various environmental compartments.^69^ However, although algal bioremediation holds promise, there are several challenges that need to be addressed to maximize its effectiveness.^70^ The technical advantages and challenges of using algal bioremediation systems are illustrated in Fig. 8.

One of the key challenges is identifying and selecting the most suitable algal species for a given pollutant. Different algal species exhibit varying capacities for pollutant uptake and degradation. Factors such as tolerance to the pollutant, growth rate, biomass production, and ease of cultivation need to be considered when choosing an algal species for bioremediation applications. Algae require specific environmental conditions to grow and thrive. Factors such as light intensity, temperature, pH, nutrient availability, and water quality play crucial roles in the performance of algal bioremediation systems. Achieving the optimal growth conditions for algae can be challenging, given that any fluctuations or imbalances in these parameters can negatively impact their growth and pollutant removal efficiency.^71^ Some emerging pollutants may exist in complex forms or be strongly bound to sediment particles, making them less bioavailable to algae. This can limit their uptake and degradation capabilities. Moreover, certain pollutants may exert toxic effects on algae, inhibiting their growth and overall performance. Thus, understanding the bioavailability and toxicity of different pollutants is essential for designing effective algal bioremediation systems. The design and scalability of algal bioremediation systems present significant challenges. The configuration of the reactors, such as open ponds, closed photo bioreactors, and wastewater treatment systems, impacts the efficiency of pollutant removal. Achieving uniform light distribution, efficient mixing, and scalable cultivation systems are important considerations for the large-scale implementation of algal bioremediation. Algal bioremediation systems should be economically viable and environmentally sustainable.^72^ The cost of algal cultivation, nutrient supplementation, monitoring equipment, and maintenance can be significant. Additionally, managing and sourcing appropriate water resources for algal cultivation is crucial, given that it can impact the sustainability of the overall process. Maintaining a consistent pollutant removal efficiency over extended periods is crucial for the success of algal bioremediation systems. Factors such as algal competition, biofilm formation, grazing by microorganisms, and changes in environmental conditions can affect the stability and long-term performance of these systems. Thus, continuous monitoring and optimization are necessary to ensure reliable and sustained pollutant removal. Addressing these challenges requires interdisciplinary research, involving expertise in algal biology, environmental engineering, chemistry, and biotechnology. Accordingly, collaborative efforts among researchers, policymakers, and industry stakeholders are essential to overcome these hurdles and advance the development and implementation of algal bioremediation systems for the effective removal of emerging pollutants.

### 6.1 Fouling

Algal bioremediation systems have gained considerable attention as a promising approach for the removal of emerging pollutants from wastewater and contaminated environments. Emerging pollutants, also known as trace organic compounds or micropollutants, include pharmaceuticals, personal care products, pesticides, and other synthetic chemicals, which are increasingly being detected in water bodies due to their persistence and widespread use. However, the successful application of algal bioremediation systems for emerging pollutant removal is often hampered by fouling challenges. Fouling refers to the undesirable accumulation of organic and inorganic substances on the algal biomass or the surfaces of the cultivation system.^73^ Fouling can lead to reduced algal growth rates, decreased pollutant removal efficiency, increased energy requirements, and system failure if not adequately addressed. One of the major fouling challenges in algal bioremediation systems is biofilm formation. Biofilms are complex microbial communities that adhere to surfaces and produce extracellular polymeric substances (EPS). These biofilms can attach to the algal cells, reactor walls, and other system components, leading to reduced light penetration, nutrient diffusion limitations, and hindered algal growth. The EP matrix can also act as a sorbent for emerging pollutants, reducing their availability for algal uptake and biodegradation. Another fouling challenge is the growth of unwanted organisms, such as bacteria and protozoa, which can compete with algae for nutrients and light. These organisms can form dense bio-flocs or settle as sediment, causing clogging and obstruction in the algal cultivation system. Additionally, the presence of grazers, such as zooplankton and snails, can consume the algal biomass, further reducing the effectiveness of pollutant removal. Physical fouling can also occur due to the accumulation of suspended solids, organic matter, or precipitated minerals. These particles can settle on the algal biomass or clog the system components, impeding light transmission and nutrient availability. Moreover, the algal cells can aggregate or flocculate, leading to sedimentation and reduced performance of the bioremediation system. Thus, to mitigate fouling challenges, various strategies can be employed, including regular monitoring and cleaning of the cultivation system, optimization of hydraulic parameters to minimize dead zones, and implementation of physical barriers or filters to prevent unwanted organisms or particles from entering the system. Furthermore, the selection of appropriate algal species with resistance to fouling and the manipulation of cultivation conditions, such as nutrient ratios and light intensity, can help mitigate fouling effects. Post-functionalization of surface membranes can be used to treat fouling.^74^ The optimal circumstances for membrane casting can be enhanced by the addition of different functional groups. The addition of functional groups to the surface, pore walls, and substructure of membranes is possible by post functionalization, a potent strategy for solving the foiling issue. Consequently, undesirable effects such as fouling on the performance of the membrane may be minimized and the surface characteristics of the membrane material can be changed to suit the needs of the application.^75^ According to a study, coating of polydopamine efficiently enhanced the wettability of polymeric membranes, while also having an anti-fouling effect.^76^ The wetting and fouling characteristics of a membrane are greatly influenced by its surface roughness. Increased surface roughness has been linked to increased fouling rates, according to another study.^77^

### 6.2 Algae biomass production rate

The successful implementation of algal bioremediation systems is associated with several challenges, particularly in terms of the algae biomass production rate. One of the primary challenges is achieving and maintaining high algal biomass productivity. The growth rate of algae is influenced by various factors, including nutrient availability, light intensity, temperature, pH, and the presence of pollutants. Thus, the optimal growth conditions must be established to ensure rapid and efficient biomass production. However, finding the ideal balance among these factors can be challenging, given that excessive nutrient concentrations can lead to eutrophication, while inadequate nutrient levels can limit algal growth. Contamination of the algal culture by emerging pollutants can also pose a challenge to biomass production.^78^ Some emerging pollutants, such as pharmaceuticals, personal care products, and pesticides, can inhibit algal growth or even exert toxic effects on the algae. Therefore, it is crucial to carefully select algal species that are resilient to the specific pollutants present in the contaminated environment. In this case, genetic engineering and selective breeding techniques can be employed to enhance the tolerance of algae towards emerging pollutants.

Furthermore, the efficient removal of emerging pollutants through algal bioremediation requires the optimization of pollutant uptake and assimilation rates. Algae employ various mechanisms to absorb and metabolize pollutants, including passive diffusion, active transport, and enzymatic degradation. However, the uptake rates of different pollutants can vary significantly, depending on their physicochemical properties and the specific characteristics of the algal species used. Enhancing the pollutant uptake efficiency and understanding the metabolic pathways involved are crucial for improving the overall remediation performance. Lastly, the scalability and cost-effectiveness of algal bioremediation systems are critical considerations.^79^ To achieve large-scale pollutant removal, it is essential to design and optimize the operational parameters, such as reactor configuration, hydraulic residence time, and biomass retention strategies. Moreover, the overall cost associated with algal biomass production, harvesting, and processing should be minimized to ensure economic viability.

### 6.3 Removal rate

The efficiency of algal bioremediation systems heavily relies on the biomass removal rate of algae, which poses several challenges. One of the primary challenges is the selection of appropriate algae species with high biomass production rates. Algae species differ in their growth rates and biomass yields, and not all species are equally effective in pollutant removal. Therefore, it is crucial to identify and cultivate algae species that have a rapid growth rate and can efficiently accumulate and degrade the targeted emerging pollutants. Maintaining the optimal environmental conditions is another critical factor affecting the biomass removal rate in algal bioremediation systems.^76^ Algae require specific conditions such as light, temperature, pH, and nutrient availability to thrive. Consequently, fluctuations in these parameters can hinder the growth and biomass production of algae, affecting the removal efficiency of pollutants. Therefore, it is essential to carefully monitor and control these environmental factors to ensure the optimal biomass production.

Inhibition of algal growth by pollutants is another challenge that can impede the biomass removal rate. Some emerging pollutants, such as heavy metals, pesticides, and pharmaceuticals, can have toxic effects on algae, inhibiting their growth and biomass production. This can lead to reduced pollutant removal efficiency and compromised overall system performance. Understanding the toxicity thresholds of different pollutants and their effects on algal growth is crucial for optimizing the biomass removal rate. Harvesting and dewatering the algal biomass is a significant challenge in algal bioremediation systems. Once the algae have accumulated pollutants, they need to be harvested and separated from the treated water. However, algae cells are typically small and dispersed, making their separation difficult. Also, traditional harvesting methods such as sedimentation, filtration, and centrifugation can be energy-intensive and costly.^80^ Thus, developing efficient and cost-effective harvesting techniques is essential to maintain a high biomass removal rate. Overall, the biomass removal rate is a critical parameter that determines the effectiveness of algal bioremediation systems for removing emerging pollutants. Overcoming the challenges related to algae selection, optimizing environmental conditions, managing pollutant toxicity, and developing efficient harvesting techniques will contribute to improving the biomass removal rate and maximizing the potential of algal bioremediation systems as a sustainable approach for environmental remediation.

## 7 New types of membranes for wastewater treatment

In addition to algal membrane bioreactors, several other membranes have also been the subject of interest in wastewater treatment research. Many methods have been developed in the past decade to create novel membranes with appropriate characteristics, such permeability, selectivity, and certain chemical and physical properties, for specific applications. Methods such as phase inversion, electrospinning, sintering, stretching, track-etching, and interfacial polymerization have been used to accomplish this. Sustainable wastewater treatment has shown significant promise after the introduction of nanomaterials and nanotechnology. The use of nanomaterials in membranes improves their mechanical strength, water permeability, separation effectiveness, and fouling reduction. Consequently, the nanomaterials open up new possibilities for incredibly quick and precise water filtration membranes. Several studies address membrane improvements after the addition of many nanomaterials. These membranes containing nanomaterials have a plethora of uses in the water treatment industry.^80^ The process of incorporating inorganic nanoparticles into polymer membranes results in the formation of embedded photocatalytic membranes. To enhance the permeability, selectivity, and physical strength of these membranes, they have undergone extensive research.^79,81^ An increasing number of water treatment applications are using more durable ceramic membranes with customizable structures and functions because of their extremely long service life and excellent mechanical, structural, chemical, and thermal stability as well as their anti-fouling qualities.^82^ This is especially true in some harsh applications. Polymer membranes based on polyvinylidene fluoride are becoming increasingly common due to their advantageous manufacturing features, high mechanical strength, and resistance to heat and chemicals.^83^ Interestingly, water treatment technologies have recently included 3D printing of a polymer membrane support or 3D printing-based interfacial polymerization. With the aid of customized and accurate 3D printing fabrication, many of the crucial characteristics can be regulated such as fouling resistance, selectivity, and water permeability.^84^ Some of the newer studies have shown that flow-electrode capacitive deionization (FCDI) offers energy-efficient and continuous desalination to remove salts from brackish water, in contrast to the membrane capacitive deionization (MCDI) technique, which has limited desalination capacity and operates in a discontinuous manner through charge/discharge cycles. To concentrate ions in one stream while desalinating the other, FCDI typically uses a suspension of powdered activated carbon (PAC) as a three-dimensional electrode that flows between the anode and cathode throughout the separation process.^85^ By using a favorable water-flux and molecular weight cut-off (MWCO) of polyethylene oxide, it has been demonstrated that hollow fiber membranes for ultra-filtration can be produced by extrusion without the need for organic solvents.^86^

## 8 Conclusion

Overall, algae-based membrane bioreactors have emerged as a promising approach for wastewater treatment due to their unique combination of algae-based treatment processes and membrane filtration. This review highlighted the physicochemical properties, advantages, and challenges associated with algae-based MBRs. The physicochemical properties of algae-based MBRs, including the use of algae as a biological component and the integration of membrane filtration, offer several advantages in wastewater treatment. Algae possess the ability to efficiently remove nutrients, such as nitrogen and phosphorus, through their natural uptake mechanisms, aiding in the reduction of eutrophication potential. Additionally, the high surface area of algae promotes the adsorption and biodegradation of organic compounds, thereby enhancing the overall removal efficiency of organic pollutants. The integration of membranes in the MBR configuration ensures excellent solid–liquid separation, enabling the production of high-quality effluent. Moreover, the produced algal biomass can be further utilized for bioenergy production or as a value-added resource, thereby offering economic and environmental benefits. However, algae-based MBRs also encounter certain challenges. One of the key challenges is the selection and cultivation of suitable algae species. The choice of algae species must be based on their compatibility with wastewater characteristics and their ability to thrive under the specific operational conditions of the MBR.

Furthermore, the efficient and stable growth of algae is highly dependent on various factors such as light intensity, temperature, pH, and nutrient availability. Maintaining these parameters within optimal ranges requires careful monitoring and control. Another challenge is the fouling of membrane surfaces by the algal biomass. The growth and accumulation of algae on the membrane can lead to reduced permeability and increased energy consumption. Effective fouling control strategies, such as pre-treatment processes, membrane cleaning techniques, and optimization of operational parameters, are crucial to mitigate fouling and maintain the long-term performance of algae-based MBRs. However, despite these challenges, algae-based MBRs offer several advantages in comparison to conventional wastewater treatment methods. They provide an environmentally friendly and sustainable approach, offering simultaneous nutrient removal and biomass production. The integration of membrane filtration ensures high-quality effluent production, meeting stringent water quality standards. Moreover, the recovery and utilization of algal biomass provide opportunities for resource recycling and energy generation. In summary, algae-based MBRs exhibit great potential for wastewater treatment due to their unique combination of algae-based processes and membrane filtration. Overcoming the challenges associated with algae selection, operational conditions, and membrane fouling will contribute to maximizing the advantages and further advancing the application of algae-based MBRs in sustainable wastewater treatment systems.

## Ethical statement

This study did not involve human participants or animals, and no ethical approval was required. All research procedures adhered to relevant ethical guidelines and best practices for non-human and non-animal research.

## Data availability

The necessary data used in the manuscript are present in the manuscript.

## Author contributions

The authors have significantly contributed to the development and the writing of this article.

## Conflicts of interest

The authors declare no conflict of interest.
